# Supplementary material for: Impact of seasonal variations in Plasmodium falciparum malaria transmission on the surveillance of pfhrp2 gene deletions
Source: eLife. 2019 May 2;8:e40339. doi: 10.7554/eLife.40339 (PMC6533063; doi:10.7554/eLife.40339)
Supplement: Transparent reporting form [file elife-40339-transrepform.pdf]

eLife's transparent reporting form

We encourage authors to provide detailed information *within their submission* to facilitate the interpretation and replication of experiments. If you have any questions, please contact us: editorial@elifesciences.org.

Sample-size estimation

- You should state whether an appropriate sample size was computed when the study was being designed
- You should state the statistical method of sample size computation and any required assumptions
- If no explicit power analysis was used, you should describe how you decided what sample (replicate) size (number) to use

Please outline where this information can be found within the submission (e.g., page numbers or figure legends), or explain why this information doesn't apply to your submission:

The outputs in the paper are model-based and so standard statistical sample size calculations do not apply. However, we have highlighted here where we justify simulation assumptions.

For all simulations carried out, 10 simulation repeats were carried with substantial population sizes equal to 100,000 (page 9). These values were considered acceptable, enabling the seasonal patterns in *pfhrp2*-negative RDTs to be observed at moderate transmission intensities (Figure 2c), while also exhibiting the stochastic variation between simulation reapeats, most noticably at the lower effective population sizes seen at lower transmission intensities (Figure 2a).

Replicates

- You should report how often each experiment was performed
- You should include a definition of biological versus technical replication
- The data obtained should be provided and sufficient information should be provided to indicate the number of independent biological and/or technical replicates
- If you encountered any outliers, you should describe how these were handled
- Criteria for exclusion/inclusion of data should be clearly stated
- High-throughput sequence data should be uploaded before submission, with a private link for reviewers provided (these are available from both GEO and ArrayExpress)

Please outline where this information can be found within the submission (e.g., page numbers or figure legends), or explain why this information doesn't apply to your submission:

For all simulations carried out, 10 simulation repeats were carried with substantial population sizes equal to 100,000 (page 9). These represent stochastic uncertainty, which at this population size is quite small. No simulations were rejected in analysis, with the population size being significantly large such that stochastic extinction of *pfhrp2* deletions did not occur

Statistical reporting

- Statistical analysis methods should be described and justified
- Raw data should be presented in figures whenever informative to do so (typically when N per group is less than 10)
- For each experiment, you should identify the statistical tests used, exact values of N, definitions of center, methods of multiple test correction, and dispersion and precision measures (e.g., mean, median, SD, SEM, confidence intervals; and, for the major substantive results, a measure of effect size (e.g., Pearson's r, Cohen's d)
- Report exact p-values wherever possible alongside the summary statistics and 95% confidence intervals. These should be reported for all key questions and not only when the p-value is less than 0.05.

Please outline where this information can be found within the submission (e.g., page numbers or figure legends), or explain why this information doesn't apply to your submission:

The paper contains mostly simulation modelling output and as such no formal statistical hypothesis tests were conducted outside of the analysis concerning the Democratic Republic of Congo (Figure 3) and Eritrea (Discussion, page 7). For figure 3, Pearson chi-squared test with Rao-Scott corrections to account for the hierarchal survey design implemented within DHS surveys were used with the p-values reported in the accompanying text on page 5, and the methods fully described on page 9. No corrections were required for Eritrea analysis.

(For large datasets, or papers with a very large number of statistical tests, you may upload a single table file with tests, Ns, etc., with reference to page numbers in the manuscript.)

Additional data files ("source data")

- We encourage you to upload relevant additional data files, such as numerical data that are represented as a graph in a figure, or as a summary table
- Where provided, these should be in the most useful format, and they can be uploaded as "Source data" files linked to a main figure or table
- Include model definition files including the full list of parameters used
- Include code used for data analysis (e.g., R, MatLab)
- Avoid stating that data files are "available upon request"

Please indicate the figures or tables for which source data files have been provided:

The simulation code is available online (see Source Code section), with the scripts and source data for the results for the online database made available within the github repository hosting the simulation code (https://github.com/OJWatson/hrp2malaRia). The online database created is also made freely available at https://ojwatson.shinyapps.io/seasonal_hrp2/.
